# Heat stress and illness among injera baking workers in Addis Ababa

**DOI:** 10.1186/s12889-026-26832-4

**Published:** 2026-03-03

**Authors:** Belay Gezahegn, Meaza Gezu Shentema, Samson Wakuma Abaya, Walelign Erkalo, Mulugeta Ayalew Eshete, Abera Kumie Takele

**Affiliations:** 1 School of Public Health, Addis Ababa University, Addis Ababa, Ethiopia; 2 Department of Public Health, Mizan Aman College of Health Science, Mizan Aman, Ethiopia

**Keywords:** Heat-related illness, Occupational heat stress, Comparative cross-sectional, Injera baking, WBGT, Ethiopia, Heat

## Abstract

Injera baking in Ethiopia involves labor-intensive work in high-temperature environments, raising concerns about occupational heat stress. Despite this, data on heat exposure and related health impacts in this setting are limited. This study aimed to assess heat exposure levels, the prevalence of heat-related illnesses (HRI), and associated factors among workers in the Injera Baking and Local Market sectors in Addis Ababa. A comparative cross-sectional study was conducted among 309 workers (159 bakers and 150 local market workers) who were selected through random sampling. Heat stress is measured by a combination of external heat and internal metabolic heat generation. Health and environmental data were collected via a modified HOTHAPS questionnaire (administered through Kobo Toolbox), supported by an observational checklist. Multivariable logistic regression was used to identify key factors associated with HRI. The median and range of WBGT during working hours at the Baking Center were 25 and 20.8–32.4 °C, respectively, higher than the comparative site, 19.5 and 13–22 °C. It exceeded ACGIH limits for moderate work from 10 AM to 4 PM at the baking center. Heat-related illness was more prevalent among bakers (46.1%) than among comparative workers (9.7%), with common symptoms including excessive sweating and fatigue. Statistically significant factors associated with HRI included workplace type (Adjusted odds ratio (AOR) = 13.2, 95% CI: 3.4–50.7), lack of cold drinking water (AOR = 4.3, 95% CI: 1.69–10.9), inadequate rest breaks (AOR = 3.5, 95% CI: 1.5–8.13), and daily water intake < 3 L (AOR = 3.9, 95% CI: 1.55–9.73). The findings underscore the urgent need for workplace interventions to reduce heat exposure and improve hydration and rest practices among Injera baking workers.

**Supplementary Information:**

The online version contains supplementary material available at 10.1186/s12889-026-26832-4.

## Introduction

Heat stress is the cumulative heat load resulting from internal metabolic heat production and external environmental sources, and it adversely affects workers’ performance, safety, and health [1]. In recent years, heat stress has become a prolonged and severe global phenomenon, intensifying with climate change and increasing the burden on various sectors, particularly in tropical and subtropical regions [2, 3]. The International Labour Organization (ILO) estimates that 2.41 billion workers (over 70% of the global workforce) are exposed to excessive heat annually, leading to approximately 22.85 million occupational injuries and nearly 18,970 heat-related deaths worldwide [4]. Excessive heat exposure can cause acute conditions such as heat rash, heat cramps, heat exhaustion, and heat stroke, and can reduce work capacity and productivity [5]. Moreover, an estimated 26.2 million people live with chronic kidney disease attributable to occupational heat stress, underscoring its long-term health burden [4]. Despite these impacts, occupational heat stress remains inadequately addressed in many countries, particularly in low- and middle-income settings [6].

The body initially responds to high temperatures through thermoregulatory mechanisms such as sweating and vasodilation [7]. However, Chronic or repeated heat exposure can overwhelm thermoregulatory mechanisms, resulting in cumulative physiological strain. Over time, workers may develop persistent dehydration, fatigue, reduced heat tolerance, and increased susceptibility to severe heat-related illnesses, including life-threatening heat stroke [8]. As climate change drives more extreme and frequent temperature events [9], certain occupations, particularly in food production and processing, are becoming increasingly vulnerable to thermal strain [10].

Many women in low- and middle-income countries (LMIC) prepare food for sale or work at small-scale marketplaces. Their work substantially contributes to the local economy, but their work environment is seldom described. Thus, this paper fills a gap and would be of interest to occupational health practitioners and researchers, as it provides important descriptive information on the workplace and work conditions. Information on workplace heat stress is also important for workplace surveillance.

Among these vulnerable occupations is injera baking, a labor-intensive and heat-intensive practice that is both economically and culturally significant in Ethiopia. Injera, a staple food for millions, is traditionally baked on hot clay griddles (mitads) that emit intense radiant heat [11]. Women, who represent the vast majority of workers in this sector, often operate in enclosed or semi-enclosed spaces with poor ventilation and limited access to cooling interventions.

Workers in these settings, such as those at the Lemi Injera Baking Center in Addis Ababa, are routinely exposed to extreme temperatures without adequate occupational safety protocols. Although studies in other sectors, such as floriculture, kitchen work, and manufacturing, have documented the impacts of heat exposure on health and productivity in Ethiopia and other low- and middle-income countries, evidence remains limited in the present study context [12]. There is a lack of published research that specifically examines occupational heat stress and heat-related illness among injera bakers. This creates a substantial evidence gap and impedes the development of effective, evidence-based interventions in this sector.

In addition, the gendered nature of this labor, being predominantly female, raises specific concerns about occupational vulnerability, especially in light of existing gender inequalities in workplace safety, access to health care, and decision-making.

This lack of targeted investigation hinders the development of effective interventions and workplace protections related to the injera baking sector. Moreover, it limits the understanding of how climate-induced occupational heat stress affects the informal. The economic and public health importance of this work highlights the urgency of research that can inform policy, promote occupational safety, and enhance the overall well-being of the workforce.

Therefore, this study aims to assess environmental heat exposure levels, the prevalence of heat-related illnesses (HRI), and associated factors among workers at the Lemi Injera Baking Center in Addis Ababa. A comparison group of local market workers was also included to contextualize the findings. By addressing this knowledge gap, the study contributes novel evidence to inform preventive strategies and occupational health improvements for injera baking workers and similar vulnerable populations.

## Materials and methods

### Study setting

Addis Ababa, the capital of Ethiopia, lies at an altitude of 2,200–2,500 m and has a temperate climate with average annual temperatures between 9.9 °C and 24.6 °C [13]. As the country’s socio-economic hub, the city contributes 38.3% of the national GDP ($61.2 billion) [14].

**Fig. 1:**
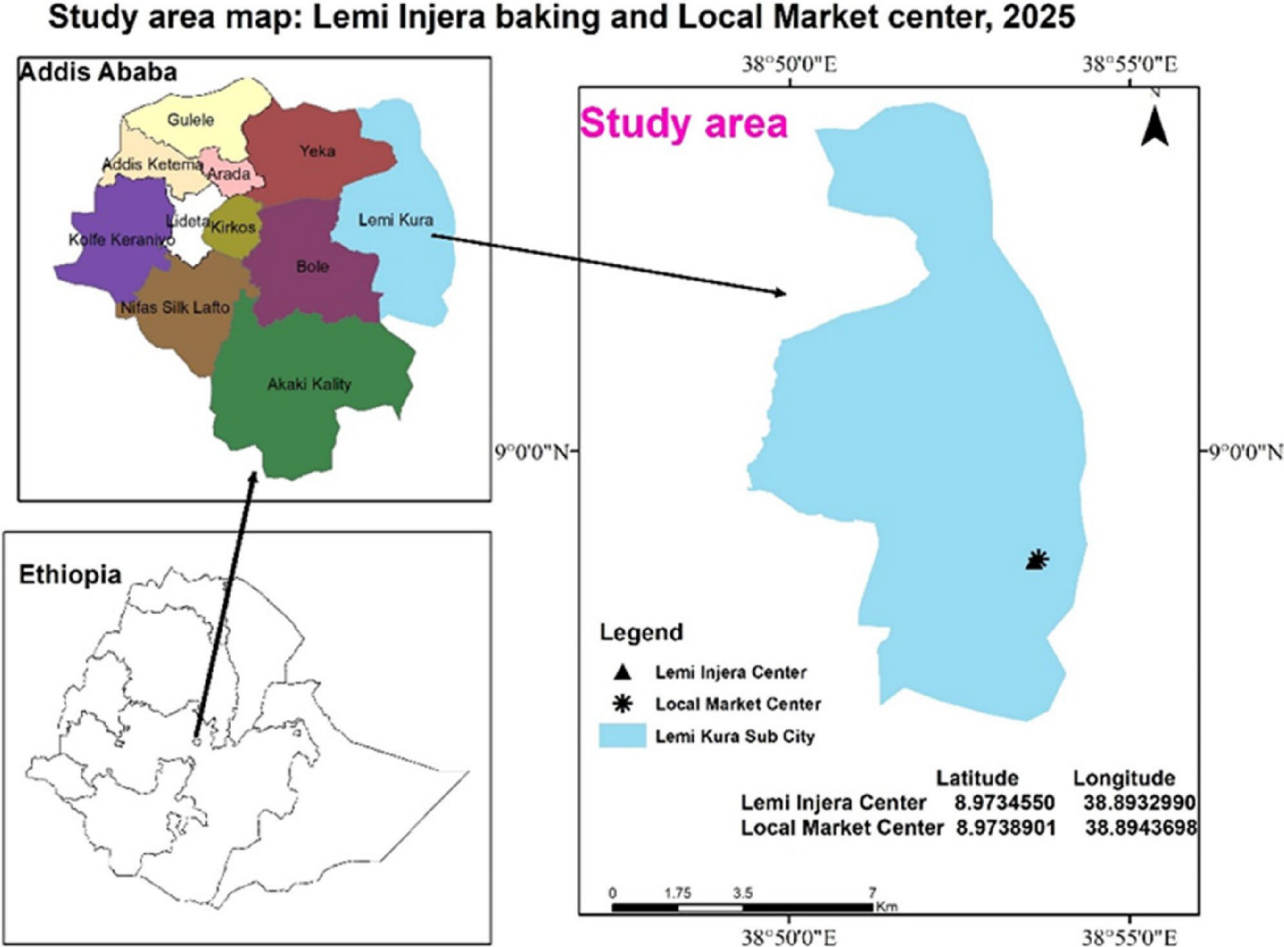
Study area: Map of Baking and Local Market, Lemi Kura, Addis Ababa, Ethiopia

The study was conducted in Lemi Kura sub-city at two sites: the Lemi Injera Baking Centre and a nearby Local Market Centre. The Injera Baking Centre, established in 2023, is a large-scale food production facility. The Local Market Centre, located within the same sub-city, serves as the comparison site due to its similar worker demographics and absence of major heat-generating activities. A map of the study area is shown in Fig. 1.

### Study design

A comparative cross-sectional study was applied to determine the level of heat exposure and HRI among workers at Lemi Injera Baking and Local Market Centers in Addis Ababa. With this design, it was possible to compare heat stress levels and HRIs between Injera baking and Market workers.

### Study period and population

Data collection was conducted from February 17 to March 2, 2025. This period was chosen because it represents one of the warmer phases in Addis Ababa’s annual climate cycle, based on data from the Ethiopian National Meteorological Agency. It reflects typical working conditions in the pre-rainy season.

The study population for this research consists of all baking workforces selected at the Lemi Injera Baking Center who experience heat stress challenges. A similar group was also selected from the Local Market workers in the nearby location.

### Inclusion and exclusion criteria

The selection of study participants began with obtaining the complete list of workers from both the Injera Baking Center and the Local Market Center. Because injera baking is performed exclusively by women, the sampling frame included all women aged 18 years and older in each site. After compiling these lists, every eligible worker was assigned an identification number, and participants were then chosen using a lottery method, allowing each woman an equal chance of being selected.

Certain groups were excluded to ensure participant safety and maintain comparability. Pregnant women were excluded because they are physiologically more vulnerable to heat stress. Likewise, women aged 60 years and older were excluded due to age-related reductions in heat tolerance.

### Sample size and sampling procedure

The final sample size for the study was 318 participants, with 159 workers from the Lemi Injera Baking Center and 159 from the Local Market Center, calculated based on double population proportion (95%CI, 90% power, 10% non-response rate, P1 = 78.2%, P2 = 61.2%: a study among hospitality kitchen workers [10]. Participants were randomly selected from a total eligible workforce of 490 injera bakers and 500 market workers using simple random sampling from their pre-prepared sampling frame. Six individuals (2 pregnant women and 4 those aged ≥ 60 years) were excluded from the study. Therefore, the sample represents a statistically powered subset, not the entire workforce.

### Data Collection

Data were collected through environmental monitoring, structured interviews, and on-site observations. Observations assessed PPE use, water access, and ventilation by data collectors. Heat exposure was measured using the QuesTemp 34WI WBGT device. WBGT heat stress measurements were conducted, four days at the baking center (two-day shifts and two-night shifts), and one day at the market center. At each site, WBGT monitoring was carried out continuously for a minimum of eight hours, with the instrument set to log measurements every five minutes. These five-minute interval readings were then averaged to produce hourly WBGT values, allowing the study to illustrate the hourly trend of heat exposure throughout the work shift.

To capture spatial variation within the workplaces, the device was positioned at three fixed locations: near the primary workstations closest to the heat source (approximately 20 cm), at the center of the production area, and at the periphery, where ventilation conditions differed. All measurements were taken at approximately 1.1 m above the ground, representing the workers’ breathing zone. Workload was assessed by observing work tasks and estimating physical activity levels in relation to task intensity and duration. Based on these observations, the workload was categorized as light, moderate, or heavy using established occupational workload classification standards.

Trained health professionals conducted interviews using a modified HOTHAPS questionnaire [15] via Kobo Toolbox to gather data on demographics, heat-related symptoms within 12 months, and work conditions. The HOTHAPS questionnaire was adapted through translation to Amharic and inclusion of context-specific symptoms such as excessive tiredness and double-layer clothing practices.

To ensure transparency and reproducibility, the original and adapted versions of the questionnaire, along with the observational checklist, have been provided as supplementary materials.

#### Operational definitions

##### Self-reported heat-related illness

Presence of ≥ 4 symptoms such as excessive sweating, fatigue, muscle cramps, headache, rashes, nausea, dizziness, or fainting during hot work periods in the last 12 months [16–18].

##### Heat stress

Total heat load from environmental and metabolic sources, assessed using WBGT per ACGIH guidelines; threshold limits are 27.5 °C for heavy and 28.5 °C for moderate workloads [19].

##### Sufficient rest break

Enough rest time excluding bathroom use > 60 min/8 h (for ≤ 8-hr work time), ≥ 75 min/8 h (for 8–10 h work time), or ≥ 90 min (for 10–12 h work time).

##### Water access

Availability of drinking water within a 3-minute walk from the work area [20].

### Data analysis

Data analysis focused on answering the main research question: What factors are associated with heat-related illness among injera baking workers compared with market workers? Descriptive statistics summarized WBGT measurements and the prevalence of heat-related illness in both groups. Median WBGT values and ranges were compared across sites to evaluate differences in heat exposure relative to occupational safety standards.

To assess associations between predictor variables and heat-related illness, we first conducted bivariate logistic regression. Variables with *p* < 0.20 were considered for multivariable analysis, not to lose important variables for risk factor assessment. Before inclusion, all candidate variables were examined for multicollinearity to ensure they did not strongly correlate with each other. A multivariable logistic regression model was then used to control for potential confounders, variables that were conceptually or statistically related to both exposure and outcome. Final covariates in the adjusted model were selected based on theoretical relevance and statistical performance.

Adjusted odds ratios (AORs) were interpreted with attention to large discrepancies between crude and adjusted estimates, acknowledging that such changes may reflect confounding or correlations among covariates. Statistical significance was set at *p* < 0.05.

### Ethical considerations

Ethical clearance was obtained from the AAU SPH Ethics Review Committee and the Addis Ababa Health Bureau. Verbal informed consent was obtained from all participants after explaining the study’s purpose, risks, and benefits. Privacy was ensured, data were securely stored, and participants with health issues were referred to nearby health centers.

## Results

### Work environment characteristics

The injera baking facility consisted of large metallic sheds constructed from thick iron sheets. Workers operated multiple electric baking stoves positioned in proximity, generating substantial radiant and ambient heat. More than 490 workers were employed in this setting. In contrast, the comparison site, a Local Market Centre within the same sub-city, housed over 500 workers preparing and selling various goods in a shaded area not exposed to heat-producing equipment. Both sites had predominantly female workers of comparable age and socioeconomic background, supporting the validity of the comparison between the two groups.

### Sociodemographic characteristics

The socio-demographic characteristics of injera bakers and market workers were largely comparable across age, education, and marital status, indicating minimal baseline differences between the two groups, as shown in Table 1 below.

**Table 1:** Sociodemographic characteristics of respondents

Variables	Categories	Work centers		*P*-value
		Injera baking N (%)	Local market	
Age category	20–34	55 (35.7%)	55 (35.5%)	0.3
	35–44	69 (44.8%)	59 (38%)	
	45–60	30 (19.5%)	41 (26.5%)	
Educational Level	No formal education	42 (27.3%)	40 (25.8%)	0.05
	Primary education	85 (55.2%)	70 (45.2%)	
	Secondary and above	27 (17.5%)	45 (29.0%)	
Marital Status	Single	9 (5.8%)	17 (11%)	0.11
	Married	120 (77.9%)	106 (68.4%)	
	Divorced	17 (11%)	16 (10.3%)	
	Widowed	8 (5.2%)	16 (10.3%)	
Availability of Cooling fans	Yes	46 (29.9%)	32 (20.6%)	0.062
	No	108 (70.1%)	123 (79.4%)	
Drinking water access	Yes	80 (51.9%)	65 (41.9%)	0.10
	No	74 (48.1%)	90 (58.1%)	
Availability of sufficient break time	Yes	72 (46.8%)	55 (35.5%)	0.05
	No	82 (53.2%)	100 (64.5%)	
Availability of a cooler work site	Yes	8 (5.2%)	66 (42.6%)	0.12
	No	146 (94.8%)	89 (57.4%)	

### WBGT-based heat exposure measurement

The measured WBGT values during working hours were consistently higher at the Baking Center compared to the Local Market site, indicating elevated heat exposure.

**Fig. 2:**
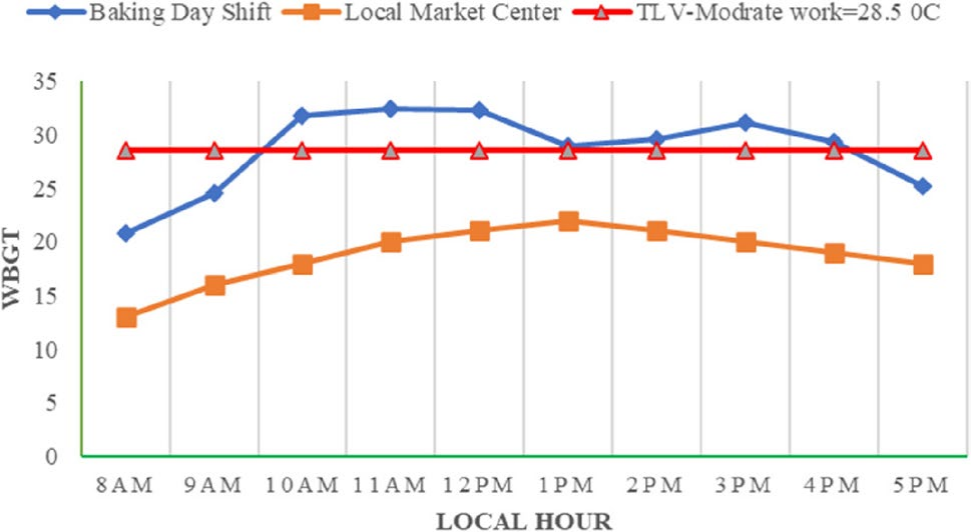
Hourly average WBGT in both work centers (TLV: Threshold limit value)

At the Baking Center, during the day shift, WBGT levels rose steadily from 20.8 °C at 8 AM to a peak of 32.4 °C at 11 AM at all sites. After a slight decrease to 32.3 °C at noon, levels dropped more notably at 1 PM, when injera baking activity was inactive at all sites. Then, after WBGT increased again to 31.1 °C at 3 PM, before gradually declined to 25.2 °C by 5 PM. Generally, these values are beyond the international standard TLV for moderate work (ACGIH) for a total of 7 h from 10 AM to 4 PM. The reference area WBGT showed consistently lower values, ranging from 13 °C at 8 AM to a peak of 22 °C at 1 PM, with more gradual changes than the Baking Center. Fig. 2 indicates generally lower heat stress at the Local Market Center during the day.

**Fig. 3:**
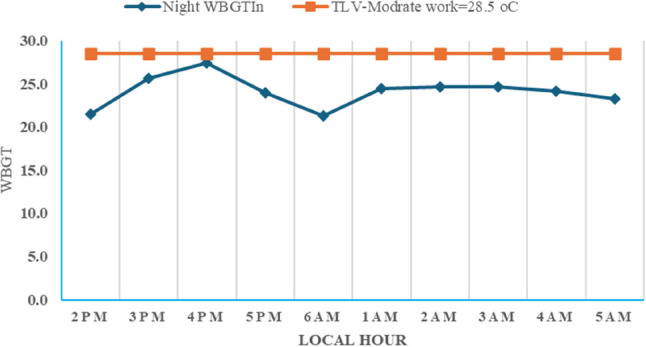
Hourly trend of WBGT in Baking Center (TLV: Threshold limit value)

During the night shift, WBGT levels increased from 8 PM and 10 PM, reaching a peak of 27.4 °C at 10 PM, when the baking activity was at its fastest with full staff. The lowest WBGT, 21.3 °C, was recorded at midnight when injera baking activity was stopped. After midnight, WBGT increased again until 3 AM as baking resumed, followed by a gradual decrease Fig. 3.

### Prevalence of self-reported heat-related illness and symptoms

Heat-related illnesses (≥ 4 symptoms) were significantly more common among Injera Baking workers (46.1%) than Market workers (9.7%), with a relative risk over 4.7 times higher. Table 2 shows that excessive sweating (85.7%) and fatigue (80.5%) were the most common symptoms among bakers.

**Table 2:** Prevalence of self-reported heat-related symptoms among study participants

Symptoms	Injera Baking	Local Market
Excessive Sweating	132 (85.7%)	44 (28.4%)
Fatigue/Exhaustion	124 (80.5%)	77 (49.7%)
Muscle Cramps	66 (42.9%)	11 (7.1%)
Headache	71 (46.1%)	19 (12.3%)
Nausea/Vomiting	53 (34.4%)	13 (8.4%)
Dizziness	24 (15.6%)	15 (9.7%)
Rapid Heartbeat	41 (26.6%)	10 (6.5%)
Confusion	21 (13.6%)	2 (1.3%)
Fainting	10 (6.5%)	-
>= 4 symptoms	71 (46.1%)	15 (9.7%)

### Multivariable logistic regression

In Table 3 Results from multivariable logistic regression showed some variables that were statistically significantly associated with heat-related illness, which include the work group, availability of cold drinking water, availability of sufficient break time, and water intake.

**Table 3:** Summary of multivariable logistic regression analysis of HRI among study participants

Variables	Category	HRIs (%)		COR (CI = 95%)	AOR (CI = 95%)	*P*-value
		Yes	No			
Work group	Injera Baking Center	71 (46.1%)	83 (53.9%)	7.98 (4.30–14.84.30.84)	13.2 (3.4–50.7)	< 0.01*
	Local Market Center	15 (9.7%)	140 (90.3%)	1.00	1.00	
Educational level	No formal education	24 (29.3%)	58 (70.7%)	2.30 (1.03–5.10)	1.4 (0.5–3.8)	0.24
	Primary education	51 (32.9%)	104 (67.1%)	2.72 (1.318–5.610)	2.1 (0.85–5.00.85.00)	
	Secondary education and above	11 (15.3%)	61 (84.7%	1.00	1.00	
Monthly income (ETB^a^)	<=2,000	5 (10.4%)	43 (89.6%)	1.07 (0.305–3.73)	1.5 (0.4–5.5)	0.83
	2,001–3,000	75 (37.5%)	125 (62.5%)	5.50 (2.26–13.39)	1.3 (0.33–5.38)	
	>=3,000	6 (9.8%)	55 (90.2%)	1.00	1.00	
Type of work	Moderate work	27 (15.4%)	148 (84.6%)	1.00	1.00	0.11
	Heavy work	59 (44.0%)	75 (56.0%)	5.91 (3.32–10.54)	1.9 (0.867–4.2.867.2)	
Working hours per day	<=8 h	17 (18.5%)	75 (81.5%)	1.00	1.00	0.53
	> 8 h	69 (31.8%)	148 (68.2%)	2.06 (1.13–3.74)	0.8 (0.35–1.7)	
Availability of Cooling fans	Yes	14 (19.7%)	57 (80.3%)	1.00	1.00	0.47
	No	72 (30.3%)	166 (69.7%)	1.77 (0.93–3.37)	1.5 (0.51–4.30)	
Drinking water access	Yes	15 (12.4%)	106 (87.6%)	1.00		0.002*
	No	71(37.8%)	117 (62.2%)	4.29 (2.32–7.94)	4.3 (1.69–10.9)	
Availability of sufficient break time	Yes	16 (14.2%)	97 (85.8%)	1.00	1.00	0.003*
	No	70 (35.7%)	126 (64.3%)	3.37 (1.84–6.16)	3.5 (1.5–8.13)	
Availability of a cooler work site	Yes	8 (10.0%)	72 (90.0%)	1.00	1.00	0.156
	No	78 (34.1%)	151 (65.9%)	4.65 (2.13–10.14)	0.5 (0.17–1.33)	
Work Clothing	Thin breathable cloth	57 (22.1%)	201 (77.9%)	1.00	1.00	0.65
	Thick cloth	29 (56.9%)	22 (43.1%)	4.65 (2.48–8.71)	1.2 (0.51–2.98)	
Water intake per day	< 3 L	8 (15.7%)	43 (84.3%)	2.33 (1.05–5.18)	3.9 (1.55–9.73)	0.004*
	>=3 L	78 (30.2%)	180 (69.8%)	1.00	1.00	

1.00 reference value, *AOR *Adjusted odds retio, *HRIs* Heat related illnesses

^a^Ethiopian Birr

*Statistically significant at *p* < 0.05; in multivariable analysis.

This study found that the odds of developing HRIs among Injera baking workers are 13 times higher than those of local market workers, AOR = 13.2 (95% CI: 3.4–50.7). Cold drinking water availability was also very important; workers without access to cold drinking water had over four times the risk of acquiring heat-related illness (AOR = 4.3, 95% CI: 1.69–10.9). Sufficient break time also had an effect; workers without sufficient breaks had over three times the risk of acquiring heat-related illness (AOR = 3.5, 95% CI: 1.5–8.13). Drinking less than 3 L of water per day during hot weather increases the odds of heat-related illness by over three times (AOR = 3.9, 95% CI: 1.55–9.73, *p* = 0.004) compared to those drinking more. Among Injera baking workers, neither BMI nor age showed a statistically significant association with HRIs (p-value > 0.05).

### Observational findings

The injera baking sites operated in enclosed rooms with multiple traditional electric and clay stoves arranged in proximity. Each worker typically managed three stoves simultaneously, working at a distance of approximately 20 cm from the heat source. Baking took place throughout the day, with most workers following long, continuous shifts depending on customer demand. The majority were self-employed or informally employed on a piece-rate basis, with payment tied to the number of injera produced.

Workload was consistently high and aligned with ISO workload classifications, corresponding to moderate to heavy physical work due to repetitive hand movements, frequent bending, and extended standing near radiant heat. Observed workloads were congruent with workers’ self-reports, which described the tasks as physically demanding and fatiguing.

The baking rooms were poorly ventilated. Although a local exhaust ventilation system existed, it was non-functional, and management reported discontinuing its use because it altered the quality. Natural ventilation was also inadequate; small openings were poorly positioned and did not align with prevailing eastern winds, resulting in limited air movement and heat accumulation.

Access to amenities was limited. Workers relied on water delivered from a mobile tanker, which was not available consistently, prompting some individuals to bring drinking water from home. Clean toilets and hand-washing areas were insufficient. Most workers wore layered work clothing, including double uniforms, which restricted heat dissipation.

Transportation to the site varied: some workers lived nearby and walked, while others relied on company transport (service bus).

The local market centers were semi-open structures with wide stalls and adequate exposure to natural airflow without any sun exposure. Ventilation was generally good, and workers performed tasks under shaded or partially covered areas. Work involved sorting, displaying, carrying goods, and interacting with customers. Based on ISO classification, vendors’ activities are aligned with light to moderate workloads.

Work hours varied, with peak customer traffic in the early morning and late evening. During the hottest hours of the day, workloads naturally reduced as foot traffic declined. This pattern matched workers’ self-reports, which described mid-day work as less labor-intensive.

Vendors had more reliable access to drinking water, often provided by nearby shops or brought from home, and typically had access to public or shared toilet facilities. Clothing was predominantly breathable cotton garments, which supported thermal comfort.

Transportation routes were diverse but generally shorter than those of the bakery workers, as markets were centrally located within residential areas.

## Discussion

The study showed differences in heat exposure level and HRIs between Injera Baking and Local Market workers in Addis Ababa. WBGT measurements showed higher heat exposure among Injera Baking workers compared to Local Market workers. The prevalence of heat-related illnesses was significantly higher among Injera Baking workers than among Local Market workers, with self-reported symptoms such as excessive sweating, fatigue, and muscle cramps being far more common among the baking group. Factors significantly associated with HRIs include work setting, access to cold drinking water, rest breaks, and water intake (*p* < 0.05).

The median WBGT during working hours at the Baking Center was 25 °C. This is in line with the study conducted in the Floriculture Industry of Ethiopia, which reported the median and range of WBGT levels were 25.5˚C and 18.1˚C to 31.5˚C, respectively [12].

In the Baking Center, WBGT peaked at 32.4 °C by 11 AM due to the combined effects of rising outdoor temperatures, sunlight, and accumulated indoor heat. A temporary drop occurred at 1 PM during break time. Between 10 AM and 4 PM, the WBGT values exceeded 28.5 °C (threshold limit) recommended by the ACGIH standard for moderate work [19]. During night shift at the Injera Baking Center, the maximum WBGT was 27.4 °C, which was around 10 PM, when baking activity was highest with full staff.

The occupational heat exposure guideline (ACGIH) is based on the maximum WBGT value for various work levels. The ACGIH standard provides specific recommendations. Based on the moderate workload and WBGT level recorded (above 28.5 °C) at the Injera Baking Center, workers should get 25% rest and 75% work per hour between 10 AM and 4 PM. As this is necessary to prevent HRI by controlling the core body temperature below 38 °C [19].

Socio-demographic characteristics of study participants were generally comparable between the two study groups, which minimizes the confounding variables when comparing HRIs. This includes the mean age, work experience, and monthly income.

The prevalence of HRIs was greatly different between the two study groups. The higher prevalence of HRIs among baking workers is linked with prolonged exposure to high-heat sources in often poorly ventilated spaces. In contrast, market workers typically operate in more ventilated settings, reducing their heat exposure risk. This result was much lower than the result from previous Ethiopian studies among other occupational settings with high heat exposure and a similar operational case definition, where HRI rates were 83.9% and 12.6%, respectively, among Steel and Pepsi-Cola factory workers [18]. Similarly, higher rates were reported among aluminum and water bottling factory workers, at 85.3% and 27%, respectively [16]. These discrepancies may be attributed to differences like heat exposure level, workload, and duration of exposure.

Among baking workers, the most commonly reported symptoms were excessive sweating and tiredness/fatigue, while fainting was rare. In contrast, market workers chiefly reported fatigue followed by sweating. These differences likely reflect the prolonged and direct exposure to radiant heat near baking stoves, which places greater thermal strain on bakers. Continuous heat exposure increases core body temperature, triggering compensatory mechanisms such as intense sweating and peripheral vasodilation to dissipate heat. Over time, this leads to dehydration, electrolyte loss, cardiovascular strain, and reduced physical endurance, explaining the higher frequency of heat-related symptoms among baking workers compared to market vendors, whose thermal exposure is intermittent and mitigated by better ventilation.

The previous study conducted in Gonder city among kitchen workers reported lower rates of sweating and tiredness, but almost similar fainting cases [10]. The variation in prevalence of the first two symptoms between the studies could be attributed to differences in work exposure time, and the larger sample size in the previous study may have influenced the detection rates of symptoms. There were significantly higher odds of developing HRIs among bakeries compared to those in the Local Market. This indicates a strong association between occupational setting and the risk of heat-related illness.

The finding was in line with the comparative study conducted in the Aluminum and Water Bottling factory near Addis Ababa, which concluded that workers in the Aluminum factory had almost ten times the odds of developing HRI compared with the control groups(AOR = 9.98, 95%CI:4.16–23.95) [16].

The absence of water access increased the odds of heat-related illness more than fourfold than those who had access to water. Other studies showed that the availability of water in a working area decreases the risk of HRIs [17, 21]. This ensures access to drinking water is a vital preventive measure against HRIs for heat-exposed workers.

The lack of sufficient rest breaks was found to be a significant risk factor in this study, with workers who did not have sufficient breaks being over three times more likely to develop heat-related illnesses. The finding was parallel with the study result from Tanzania and Japan, which highlights that the absence of sufficient breaks was associated with a higher risk of HRIs [21, 22]. This shows the importance of routine rest periods as an effective measure to reduce HRI risks in hot working environments.

Concerning water intake, workers who consumed less than 3 L of water per day had over two times the odds of developing heat-related illnesses compared to those who drank 3 L or more. The National Institute for Occupational Safety and Health (NIOSH) recommends a daily fluid intake of 5.7 to 9.5 L for individuals working in hot environments. Inadequate water intake impairs the ability of body to regulate temperature, which increases the risk of HRIs. The finding is nearly supported by research conducted among industrial workers that found individuals consuming less than 3 L of water per day were five times more likely to experience HRIs compared to those taking 3 L or more [16]. A study from Malaysia also reported consistent results, reinforcing the importance of adequate fluid intake in preventing HRIs for heat-exposed workers [23].

Neither BMI nor age showed a statistically significant association with HRIs (p-value > 0.05). This is supported by a study done in Tanzania [21]. However, other studies have reported that obese individuals have a higher risk of developing HRI than those with normal BMI [24, 25], which is contrary to the finding here. This is due to the fact that workers with higher BMI in this study may have been less physically active.

These findings highlight the need for interventions such as adding more work-rest scheduling, water access, and worker education on HRI risks to mitigate them in the workplace.

The study’s strengths include the use of WBGT monitoring, robust statistical modeling, and a comparative design enhancing internal validity. However, this study has several limitations. First, heat-related illness symptoms were self-reported, which may be subject to recall bias and social desirability bias, potentially leading workers to under- or over-report symptoms based on personal perceptions or workplace expectations. Second, the study population consisted exclusively of women in informal and gender-segregated occupations, meaning that factors such as caregiving roles, economic vulnerability, or job insecurity may have influenced both exposure and symptom reporting. Finally, because the research was conducted in a single urban setting and within a specific industry, the findings may not be generalizable to other regions, climates, or occupational groups.

## Conclusions

Injera Baking workers were exposed to significantly higher heat levels than Local Market workers. The WBGT values at the baking center often exceed the ACGIH TLV of 28.5 °C for moderate work, particularly between 10 AM and 4 PM. Of baking workers, 46.1% had reported HRI, and 9.7% among the Market Center workers. The associated factors with HRI include limited access to drinking water, fewer rest breaks, and inadequate water intake. The findings underscore the urgent need for targeted interventions to minimize heat exposure and enhance workplace safety in baking environments.

## Supplementary Information

Supplementary Material 1.

## Data Availability

The datasets used and/or analyzed during the current study are available from the corresponding author upon reasonable request.
