# Supplementary material for: Heat stress and illness among injera baking workers in Addis Ababa
Source: BMC Public Health. 2026 Mar 3;26:1153. doi: 10.1186/s12889-026-26832-4 (PMC13063609; doi:10.1186/s12889-026-26832-4)
Supplement: Supplementary file 1 — Supplementary Material 1. [file 12889_2026_26832_MOESM1_ESM.docx]

## **Annex I: Data Collection Tools**

### **English version Questionnaire**

Name of the interviewer: ______________

Form ID: __________ Date: __________ Factory: _________

**Table 1: English version Questionnaire for Heat Stress Exposure Assessment Study.**

| **Parts** | **Questions** | **Responses** | **Remark** |
| --- | --- | --- | --- |
| **Part I: Identifiers** | 1. Interviewer ID |  |  |
|  | 1. Form ID |  |  |
|  | 1. Date and time |  |  |
| **Part II: Demographic Information** | 1. In which section are you working? | 1. Batter preparation 2. Baking 3. Storage 4. Market center |  |
|  | 1. How old are you? (In years) | __________years |  |
|  | 1. Education Level | 1. No formal education 2. Primary education 3. Secondary education 4. College/ University 5. Other   (specify) _________ |  |
|  | 1. What is your Marital Status? | 1. Single 2. Married 3. Divorced 4. Widowed |  |
|  | 1. Your average monthly income | _______ETB |  |
| **Part III: Physical measurement and NCD Risk factors** | 1. Body Weight | ______Kg |  |
|  | 1. Body Height | ______m |  |
|  | 1. BMI | ______Kg/m^2^ |  |
|  | 1. Systolic BP | ______mmHg |  |
|  | 1. Diastolic BP | ______mmHg |  |
|  | 1. Body temperature | ______^0^C |  |
|  | 1. Smoking hx | 1. Smoker 2. Non-Smoker 3. Ex-smoker |  |
|  | 1. History of alcohol consumption | 1. Yes, regularly 2. Yes, sometimes 3. Not at all 4. Previous drunker |  |
|  | 1. Do you have any chronic disease confirmed by a health professional? | 1. Yes 2. No |  |
|  | 1. If yes to Q#17, which medical illness do you have? | 1. Diabetes 2. Hypertension 3. Respiratory illness, specify ________ 4. Kidney disease   Others, specify_________ |  |
| **Part IV: Occupation-related** | 1. Do you have a previous job? | 1. Yes 2. No |  |
|  | 1. If yes to Q#19, what was your previous job? | 1. __________________ |  |
|  | 1. How long have you been working at the center? | _______ years /month |  |
|  | 1. Level the type of work you are currently doing | 1. Light work 2. Moderate work 3. Heavy work |  |
|  | 1. Are you comfortable with the workplace temperature? | 1. Yes 2. No |  |
|  | 1. On a scale of 1 to 5, how would you rate the heat levels in your workplace? | 1. Very low 2. Low 3. Moderate 4. High 5. Very high |  |
|  | 1. Do you work near direct heat? | 1. Yes 2. No |  |
|  | 1. Which shift do you usually work? | 1. Morning 2. Afternoon 3. Night 4. Rotating shifts 5. No shift (full day) |  |
|  | 1. How many hours do you work without break time per day on average? | __________hours |  |
|  | 1. Working in a hot environment affects which of these? | 1. Health 2. Work focus 3. Productivity 4. Other (specify) _______ |  |
| **Part V: Health Symptoms and Conditions** | 1. Have you ever felt dizzy, fatigued, or nauseous due to heat while working? | 1. Yes 2. No |  |
|  | 1. If yes, how often do you feel these symptoms? | 1. Frequently 2. Occasionally 3. Rarely 4. Never |  |
|  | 1. Have you ever reported symptoms of heat stress to your supervisor? | 1. Yes 2. No |  |
|  | 1. If yes, what actions were taken? | 1. Take a break 2. Replace the worker 3. No action taken 4. Other   (specify)_____________ |  |
|  | 1. Have you experienced any of the following symptoms while at work (hot day) in the last 12 months? (Select all that apply.) | 1. Excessive sweating 2. Fatigue or exhaustion 3. Muscle cramps 4. Headache 5. Nausea or vomiting 6. Dizziness 7. Fainting 8. Rash (Skin, chest, and neck redness) 9. No |  |
|  | 1. Have you ever been diagnosed with heat-related illnesses in the health facility? | 1. Yes 2. No |  |
| **Part VI: Coping Mechanisms and consequences of Heat stress** | 1. What measures do you personally take to cope with the heat at work? (Select all that you apply): | 1. Drink more water 2. Take more breaks 3. Wear lighter clothing 4. Use a fan or other cooling device 5. Other   (specify): _______ |  |
|  | 1. Does the company provide any of the following to reduce heat stress? (Select all that apply): | 1. Cooling fans 2. Cold water 3. Extra breaks 4. Cooler workstations 5. Other   (specify): _______ |  |
|  | 1. Which kind of PPE could you use? | 1. Gauntlet gloves 2. Coverall 3. Facemask 4. Google 5. Other PPE   (specify)_________ |  |
|  | 1. Approximately how many liters of water do you drink per day in hot time? | _______L |  |
|  | 1. What kind of cloth do you mostly wear during your work? | 1. Breathable cotton 2. Thick cotton overall 3. Rayon/Nylon 4. Plastic PPE 5. Others_________ |  |
|  | 1. Does the cloth exacerbate heat? | 1. Yes 2. No |  |
|  | 1. Have you got any training on heat stress? | 1. Yes 2. No |  |
|  | 1. If yes when? | 1. Not more than 6 months 2. 6 months- 1year 3. More than 1 year |  |
|  | 1. Have you ever taken sick leave/permission due to heat? | 1. Yes 2. No |  |
|  | 1. If yes, question 39 | How many days ____________ |  |
|  | 1. Have you ever been hospitalized for heat-related illness? | 1. Yes 2. No |  |
|  | 1. If yes to question 46 | Approximately how many days _________ |  |
|  | 1. Have you lost wages or salary due to heat-related illness? | 1. Yes 2. No |  |
|  | 1. If yes to question 47, how much | _____________birr |  |

### **Amharic version of Questionnaire**

የጠያቂው ስም፡- ______________

የቅጹ መለያ ቁጥር፡_________ ቀን፡ __________ የስራ ቦታ፡ __________

**Table 2: Amharic version Questionnaire for Heat Stress Exposure Assessment Study.**

| **ክፍሎች** | **ጥያቄዎች** | **ምላሾች** | **ምርመራ** |
| --- | --- | --- | --- |
| **ክፍል አንድ፡ መለያዎች** | 1. የጠያቂው መለያ | **___________________** |  |
|  | 1. የቅጹ መለያ ቁጥር | **_______________** |  |
|  | 1. ቀንና ሰዓት | **______________** |  |
| **ክፍል ሁለት፡ የስነ ሕዝብ አወቃቀር መረጃ** ክብደት | 1. በየትኛው የስራ ቡድን ነው የምትሰሪው? | 1. ሊጥ ዝግጅት 2. በእንጀራ መጋገር 3. በእንጀራ ማከማቻ 4. ገበያ ማዓከል |  |
|  | 1. እድሜሽ ስንት ነው? (በአመት) | _________ |  |
|  | 1. የትምህርት ደረጃ | ሀ) መደበኛ ትምህርት የለም  ለ) የመጀመሪያ ደረጃ ትምህርት  ሐ) የሁለተኛ ደረጃ ትምህርት  መ) ኮሌጅ / ዩኒቨርሲቲ  ሠ) ሌላ (ይግለጹ)_________ |  |
|  | 1. የጋብቻ ሁኔታሸ? | ሀ) ያላገባ  ለ) ያገባ  ሐ) የተፋታ  መ) ባል የሞተባት |  |
|  | 1. የእርስዎ አማካይ ወርሃዊ ገቢ | ______________________*ኢትዮጵያ ብር* |  |
| **ክፍል ሶስት፡ አካላዊ ልከት እና ተላላፊ ላልሆኑ በሽታዎች አጋላጭ ሁኔታዎች** | 1. ክብደት | ------ሜትር |  |
|  | 1. ቁመት | -------- ኪ.ግ |  |
|  | 1. የሰውነት ክብደት መረጃ ጠቋሚ(BMI) | ________________ኪግ/ሜ^2^ |  |
|  | 1. ሲስቶሊክ ቢፒ (mm Hg) | __________________ mm Hg |  |
|  | 1. ዲያስቶሊክ ቢፒ | _________________ mm Hg |  |
|  | 1. የሰውነት ሙቀት | ____________________^0^C |  |
|  | 1. ስጋራ አጭሰዉ ታዉቅያለሽ | 1. አዎ(አጫሽ) 2. በፍጹም አላጨስኩም 3. ድሮ አጨስ ነበር |  |
|  | 1. አልኮል መጠጥ ትጠጣለሽ? | 1. አዎ 2. አይ በፍጹም 3. የቀድሞ ጠጪ |  |
|  | 1. በጤና ባለሙያ የተረጋገጠ ሥር የቆየ በሽታ (ተላላፍ ያልሆኑት) አለብሽ? | 1. አዎ 2. አይ |  |
|  | 1. ጥያቄ ቁ 17 አዎ ከሆነ የትኛው? (ብዙ መልስ መምረጥ ይቻላል)? | 1. የስኳር በሽታ 2. የደም ግፊት 3. የአተነፋፈስ ሕመም፣ ________ ይግለጹ 4. የኩላሊት በሽታ 5. ምንም የለኝም 6. ሌሎች፣ ይግለጹ________ |  |
| **ክፍል አራት፡ ከስራ ጋር የተያያዘ** | 1. ቀድሞ ለላ ሥራዎ ነበረሽ? | 1. አዎ 2. አይደለም |  |
|  | 1. ጥያቄ ቁ 19 አዎ ከሆነ ስራዉ ምን ነበር? | _________________________________ |  |
|  | 1. በዚህ ማእከል ምን ያህል ጊዜ እየሰሩ ነው? | ____________________ዓመት |  |
|  | 1. የሥራውን ክብደት እንደት ትገልጻልሽ? | 1. ቀላል ሥራ 2. መጠነኛ ሥራ 3. ከባድ ሥራ |  |
|  | 1. በሥራ ቦታ የሙቀት መጠን ምቾት ይሰማዎታል? | 1. አዎ 2. አይደለም |  |
|  | 1. ከ 1 እስከ 5 ባለው ልኬት፣ በስራ ቦታዎ ያለውን የሙቀት መጠን እንዴት ይመዝኑታል? | 1. በጣም ዝቅተኛ 2. ዝቅተኛ 3. መካከለኛ 4. ከፍተኛ 5. በጣም ከፍተኛ |  |
|  | 1. በቀጥታ ሙቀት አጠገብ ይሰራሉ? | 1. አዎ 2. አይደለም |  |
|  | 1. አብዛኛዉን ጊዜ የምትሠሪው በትኛውን ፈረቃ ነው? | 1. ጥዋት 2. ከሰዓት በኋላ 3. ምሽት 4. የሚሽከረከር ፈረቃ 5. ፈረቃ የለም (ሙሉ ቀን) |  |
|  | 1. በቀን በአማካይ ምን ያክል ሰአታትን ያለ እረፍት ሰአት ይሰራሉ? | ________________________ሰአታት |  |
|  | 1. በሞቃት አካባቢ ውስጥ መሥራት ምን ዓይነት ተጽእኖ ይኖረዋል? | 1. በጤና ላይ ተጽእኖ አለው 2. ትኩረት በመስጠት ላይ ተጽእኖ ይኖረዋል 3. በምርታማነት ላይ ተፅእኖ አለው 4. ሌላ ይግለጹ________________________ |  |
| **ክፍል አምስት፡ የጤና ምልክቶች እና ሁኔታዎች** | 1. በሥራ ላይ እያለሽ በሙቀት ምክንያት የማዞር፣ የድካም ወይም የማቅለሽለሽ ስሜት ተሰምቶህ ያውቃል? | 1. አዎ 2. አይደለም |  |
|  | 1. አዎ ከሆነ፣ እነዚህ ምልክቶች ምን ያህል ጊዜ ተሰምቶሻል? | 1. በተደጋጋሚ 2. አልፎ አልፎ 3. አንዳንደ 4. በጭራሽ |  |
|  | 1. የሙቀት ጭንቀት(heat stress) ምልክቶችን ለተቆጣጣሪዎ ሪፖርት አድርገው ያውቃሉ? | 1. አዎ 2. አይደለም |  |
|  | 1. ጥ.ቁ32 አዎ ከሆነ ምን እርምጃ ተወሰደ? | 1. እረፍት ተሰጠኝ 2. ለላ ሰው ተተካ 3. ምንም 4. ለላ ይገለጽ ______________________________ |  |
|  | 1. በስራ ላይ እያሉ ከሚከተሉት ምልክቶች አንዱን አጋጥመውዎታል? (የሚመለከተውን ሁሉ ይምረጡ) | 1. ከመጠን በላይ ላብ 2. ድካም ወይም ድካም 3. የጡንቻ ሕመም 4. ራስ ምታት 5. ማቅለሽለሽ ወይም ማስታወክ 6. መፍዘዝ 7. ፈጣን የልብ ምት 8. ግራ መጋባት 9. ራስን መሳት 10. የቆዳ፣ የደረት እና የአንገት መቅላት 11. ከላይ ከተጠቀሱት ውስጥ አንዳቸውም አይደሉም |  |
|  | 1. ከሙቀት ጋር በተያያዙ ህመሞች እንዳለዎት በጠና ባለሙያ ተነግሮት ያውቃል? | 1. አዎ 2. አይደለም |  |
| **ክፍል ስድስት፡ የመቋቋሚያ ዘዴዎች እና የሙቀት ውጤቶች** | 1. በስራ ላይ ያለውን ሙቀት ለመቋቋም በግል የሚወስዷቸው እርምጃዎች የትኞቹ ናቸው? (የሚመለከተውን ሁሉ ይምረጡ) | 1. ብዙ ውሃ መጠጣት 2. ተጨማሪ እረፍት ማድረግ 3. ቀላል ልብስ መልበስ 4. የአየር ማራገቢያ ወይም ሌላ ማቀዝቀዣ መሳሪያ መጠቀም 5. ሌላ (ይግለጹ)፡_____________ |  |
|  | 1. የሚመለከተው አካል የሙቀት ጭንቀትን ለመቀነስ ከሚከተሉት ውስጥ የትኛውን ያቀርባል? (የሚመለከተውን ሁሉ ይምረጡ) | 1. የአየር ማናፈሻ/ ማቀዝቀዣ 2. ቀዝቃዛ ውሃ 3. ተጨማሪ እረፍቶች 4. ቀዝቃዛ የሥራ ቦታዎች 5. ሌላ (ይግለጹ) |  |
|  | 1. ምን አይነት የግል መከላከያ መሳሪያ ነው የምትጠቀመው? | 1. ጋውንትሌት ጓንቶች 2. ሙሉ ሽፋን 3. የፊት ጭንብል 4. ጎግል 5. ሌላ (ይግለጹ)__________________ |  |
|  | 1. በሞቃት ጊዜ በግምት በቀን ምን ያህል ሊትር ውሃ ይጠጣሉ? | ___________ሊ |  |
|  | 1. ​​በስራዎ ወቅት ምን አይነት ልብስ ይለብሳሉ? | 1. አየር የሚፈቅድ ጥጥ / ቀላል ልብስ 2. በአጠቃላይ ወፍራም ጥጥ 3. ሬዮን/ናይሎን 4. የፕላስቲክ የግል መከላከያ መሳሪያዎች 5. ሌሎች_____________________ |  |
|  | 1. ልብሱ ሙቀትን ያባብሳል? | 1. አዎ 2. አይደለም |  |
|  | 1. በሙቀት ጭንቀት(heat stress) ላይ ምንም ዓይነት ስልጠና አግኝተሽ ታውቅያለሽ? | 1. አዎ 2. አይደለም |  |
|  | 1. ጥ.ቁ41 አዎ ከሆነ መቼ? | ________________ |  |
|  | 1. ባለፈው አንድ አመት ውስጥ በሙቀት ምክንያት የሕመም እረፍት/ፈቃድ ወስደሽ ታውቃለሽ? | 1. አዎ 2. አይደለም |  |
|  | 1. ለጥያቄ 43 መልስዎ አዎ ከሆነ ስንት ቀናት? | ____________ቀናት |  |
|  | 1. ከሙቀት ጋር በተያያዙ በሽታዎች ሆስፒታል ገብተው ተኝተው ያውቃሉ? | 1. አዎ 2. አይደለም |  |
|  | 1. ለጥያቄ 45 መልስዎ አዎ ከሆነ | በግምት ስንት ቀናት _________ |  |
|  | 1. ከሙቀት ጋር በተገናኘ ህመም ምክንያት ደሞዝ አጥተዋል? | 1. አዎ 2. አይደለም |  |
|  | 1. ለጥያቄ 47 መልስዎ አዎ ከሆነ | _____________ኢት ብር |  |

### **Checklist for observation**

**Table 11: Checklist for Heat Stress Exposure Assessment Study.**

| **Items** | | **Yes** | **No** | **Comment** |
| --- | --- | --- | --- | --- |
| Working environment (Ventilation) | Is the workplace well-ventilated? |  |  |  |
|  | Are open doors and windows used to increase airflow? |  |  |  |
|  | Are fans used in poorly ventilated areas to enhance air circulation? |  |  |  |
| PPE access | Do workers wear thin, air-permeable clothing? |  |  |  |
|  | Does the job require the use of heat-protective clothing? |  |  |  |
| Water and rest facility | Is drinking water available in locations close to all workers? |  |  |  |
|  | Are sufficient rest or break areas equipped with cooling rooms? |  |  |  |
|  | Are showers readily accessible when needed? |  |  |  |
